# Quality improvement for cancer multidisciplinary teams: lessons learned from the Anglian Germ Cell Cancer Collaborative Group

**DOI:** 10.1038/s41416-020-01080-4

**Published:** 2020-09-29

**Authors:** Tayana Soukup, Nick Sevdalis, James S. A. Green, Benjamin W. Lamb

**Affiliations:** 1 Center for Implementation Science, Health Service and Population Research Department, King’s College London, London, UK; 2 Whipps Cross University Hospital, Barts Health NHS Trust, London, UK; 3 Department of Urology, Cambridge University Hospitals NHS Foundation Trust, Cambridge, UK

**Keywords:** Germ cell tumours, Health services

## Abstract

Shamash and colleagues describe how their supra-regional germ cell tumour multidisciplinary team achieved standardisation of treatment and improved survival. We discuss some of the insights the study provides into prioritising complex patients, streamlining processes, the use of telemedicine, and the centrality of good data collection to continuous quality improvement.

## Main

In the months before the coronavirus disease 2019 (COVID-19) pandemic, multidisciplinary team (MDT) working in cancer care was again on the stakeholder’s agenda. Interest had been piqued with the Gore report,^1^ which called for a change in the way MDTs worked to meet the challenges of increasing workload, while maintaining quality. Since the 1990s, the United Kingdom’s system of MDT working with networks of MDTs and cancer centres (and for rare tumours, of supra-network MDTs) has become the gold standard and a model for other countries.

Shamash and colleagues^2^ describe the impact of a supra-network MDT (SMDT) on clinical decision-making and outcomes for patients with germ-cell tumours in the east of England.

Although all patients with a diagnosis of germ-cell tumour were discussed at the MDT meeting, patients with favourable features were discussed briefly, permitting more time for discussion of complex cases. Patients needing emergency treatment were discussed with the chair outside the meeting to ensure timely initial therapy. MDTs across the region met weekly via video conferencing. Oversight was provided through annual governance meetings, which included reviews of operational policy and clinical trials. Importantly, a standardised proforma was developed and used for submission of patient details to the SMDT, which also formed the basis of data submitted for their manuscript.^2^

Between 2007 and 2017, 2892 new cases of germ-cell tumour were reviewed by the SMDT. Although changes to radiological and pathological reports and recommendations for patient management were seldom made (3, 5.4, and 6.4%, respectively), other benefits of the supra-regional network are apparent.^2^

Overall survival at lower-volume centres (87.8–93.3%) increased to that of higher-volume centres (95%). Improvements arose from standardisation of chemotherapy regimens, increased adoption of novel regiments, and more equitable access to such treatments. 18-Fluorodeoxyglucose positron emission tomography/computed tomography was found to be less useful than previously described. An additional benefit of the SMDT was as a focus to improve the outcome of smaller centres as an alternative to further centralisation of services.^2^

The data presented by Shamash and colleagues^2^ also gives an insight into the organisation of a complex but well-functioning network covering numerous sites and different specialists over a significant geographical location. This group had put into practice the suggestions set out in the Gore report^1^ years ahead of publication.

Taking each of these individually:

### Focussing on complex cases

The greatest benefit of MDT working is seen in complex cases, e.g. unusual subtype of disease, failure of previous treatment, significant comorbidities, and social or psychological problems.^2^ These patients often do not fit guidelines, are not eligible for clinical trials, and can be challenging to engage in healthcare services. Shamash et al.^2^ highlight patients with learning difficulties or mental health problems and those with late relapses each present problem that are less commonly addressed and require tailored individualised treatment plans. These findings are in line with the recent study on what constitute a complex case for MDT discussion, mirroring those found to be indicators of complexity across a range of tumour types.^3^ Although they represent a small portion of cases, considerable amount of additional support is needed before and after diagnosis and treatment.^2^

Shamash and colleagues^2^ set out criteria for cases that may not need full discussion in the MDT meeting. It may be desirable to go further and identify cases that are truly ‘complex’ and those that are ‘simple’. Recently, Soukup and colleagues^3^ published work on the development and validation of a tool for stratifying cases by complexity, which might allow teams to streamline their caseload in a scientific manner. Further research is needed to assess its impact on patient care and the efficiency of MDT processes.

The inclusion of information on patients’ comorbidities and psychological and social factors that may impact care are persistently, poorly represented in MDT meetings.^4^ Such information, as well as that which focusses on the disease in question, is necessary for comprehensive clinical management planning.^5^ These findings support the conclusions by Shamash and colleagues,^2^ that patients with complicating features require holistic discussion in order to develop tailored treatment plans.

### Using chair’s action to facilitate urgent treatment

The time between meetings can present a significant period for patients with rapidly progressing disease waiting for MDT review and recommendations.^2^ In such cases, the MDT chair is well placed to endorse management proposals of clinicians out with the MDT meeting in order to avoid delays.^2^ Such cases should still be registered with the MDT and could be reviewed post hoc. The responsiveness of an MDT to clinical or organisational pressures is an area fertile for improvement.

### The use of videoconferencing to improve collaborative decision-making

Videoconferencing has been controversial in MDT meetings, and Shamash and colleagues^2^ discuss some of its advantages and challenges. Regular SMDT meetings are not feasible without some form of remote contact.^2^ Technology failure and differences in communication styles can present challenges to the quality of MDT decision-making.^6^ Perhaps a lasting legacy of COVID-19 will be the dramatic shift towards telemedicine, replacing many face-to-face interactions. Interestingly, Shamash and colleagues^2^ note the benefits of a yearly meeting at which members of the SMDT can interact and discuss matters of importance. Many MDTs now manage to operate remotely via video link. It may be desirable to supplement this with periodic face-to-face interaction that permit more nuanced communication regarding performance, operational policy, challenges, and future directions.

### Data collection and audit

The careful, planned collection of clinical and process data was crucial for assessing complex areas of healthcare, such as care pathways and organisational changes.^2^ Recent NHS England and NHS Improvement report^7^ has highlighted that data collection and regular audit must accompany MDT transformation. As Shamash and colleagues^2^ showed, the collection and analysis of such data might provide a resource to benchmark processes and outcomes, thereby driving standardisation and convergence towards best practice. Well-designed data collection supports quality improvement and clinical research, driving the development of new and better standards of care. Ultimately, this will provide high-quality information to patients and their doctors, enabling shared decision-making of the highest quality.

## Data Availability

Not applicable.
